# Poverty, Somatisation Tendency and Potency in Low-Income Adolescent Groups of India and Israel: Explorations from the Field

**DOI:** 10.3390/children10071104

**Published:** 2023-06-23

**Authors:** Saoni Banerjee, Rachel Lev-Wiesel, Sonali De

**Affiliations:** 1 Emili Sagol Research Center for CAT (Creative Arts Therapies), University of Haifa, Haifa 3498838, Israel; rlev@univ.haifa.ac.il; 2 Body & Mind Psychotherapy Track, Social Work, Tel Hai Academic Center, Qiryat Shemona 1220800, Israel; 3 National Center for Children at Risk Assessment, The Sagol Center for Hyperbaric Treatment and Research, Shamir Hospital, Be’er Ya’akov 60930, Israel; 4 FAA-Emili Sagol Creative Arts Research and Innovation for Well-Being Center at Chulalongkorn University (CARIW), Bangkok 10330, Thailand; 5 Department of Psychology, Calcutta University, Kolkata 700009, India; sonalide2002@yahoo.com

**Keywords:** somatisation tendency, potency, adolescent, perception of poverty, relative poverty

## Abstract

Poverty increases vulnerability towards somatisation and influences the sense of mastery and well-being. The present study on adolescents living in relative poverty in a high-income group country (Israel) and a low-middle-income group country (India) explored the nature of somatisation tendency (ST) and its relationship with potency and perception of poverty (PP). Potency, a buffer against stress-induced negative health effects, was hypothesized to be negatively related to ST and mediate the link between PP and ST. Purposive sampling was used to collect questionnaire-based data from community youth (12–16 years) of two metropolitan cities—Kolkata (India, N = 200) and Tel-Aviv (Israel, N = 208). The nature of ST, PP and potency was analysed using descriptive and inferential statistics and correlation-regression statistics and mediation analysis were used to understand the relationship among them. A clinically significant level of ST was reported by both Indian and Israeli youth experiencing 5–7 somatic symptoms on average. Potency was found to be a significant predictor of ST in both countries (*p* < 0.05) and emerged as a significant mediator (*p* < 0.001) in the PP and ST relationship among Indian adolescents. The present study highlights potency as a protective buffer in economically vulnerable community adolescents and re-establishes a high prevalence of ST among them, irrespective of their country’s global economic position.

## 1. Introduction

**Figure 1:**
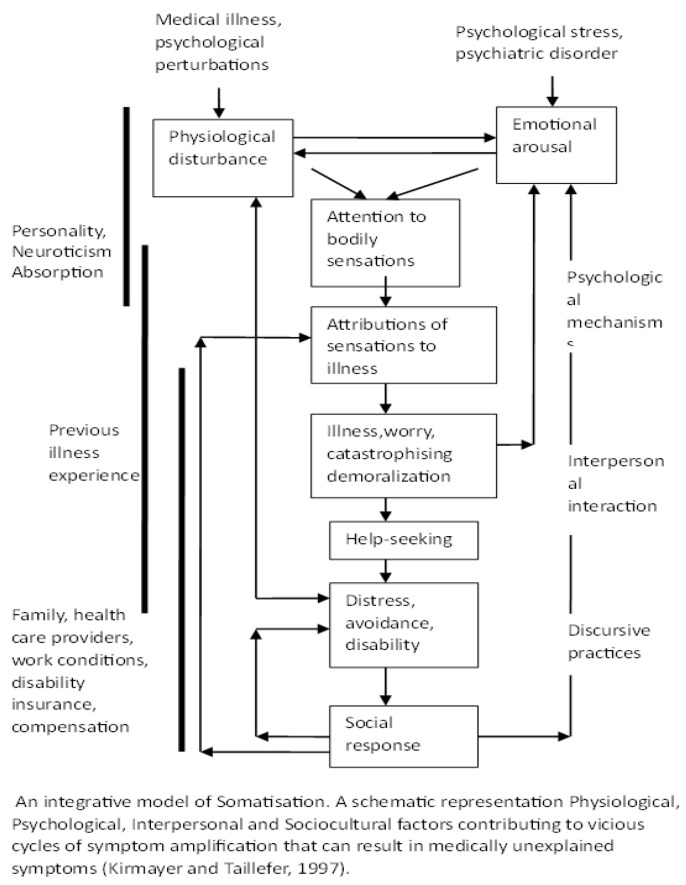
An integrative model of somatisation [6].

Somatic symptoms are purported manifestations of physical illness accompanied by heightened awareness of certain bodily sensations [1]. It is found to be high among adolescents globally [2,3], with a comparatively lower prevalence in Western populations [4] and males [5] compared with Eastern populations and females, respectively. Study on the contributing factors has reigned in the research arena in comparison to limited studies on preventive psychological resources. An integrative schematic model is reproduced here (Figure 1) to highlight the already established sociocultural and physiological factors contributing to somatisation, symptom amplification and distress [6]. Psychological resources are the protective determinants of health and well-being [7], especially during transitional periods [8]. They can be interpersonal, i.e., those generated through one’s relationship with society, and intrapersonal, or those influenced by one’s inherent nature and experiences.

## 2. Background

### 2.1. Somatisation Tendency

Somatisation is the process of ‘transforming’ one’s psychological conflict into bodily symptoms and/or somatic preoccupation in the absence of an identifiable organic cause [9]. Clinical understanding of ‘somatisation’ or ‘somatic symptom’ has seen a shift in both terminological and theoretical approaches, moving from a causation-centric approach (whether-or-not medically attributable to or associated with an established organic condition) to being effect-centric (severity of experienced impairment or distress) and symptom-centric (the organic system or areas of impairment). Somatisation tendency (ST) here is defined as the propensity (not a formed disorder) to report distress causing somatic symptoms, irrespective of whether it can be fully explained by the currently available biomedical assessments upon investigation. Long-drawn debates around the diagnostic criteria of somatisation have led to the formation of a new label, ‘somatic symptom disorder’ (SSD), in the current diagnostic and statistical manual for mental health disorders (DSM-V). The new diagnosis focused on the temporal extensity and perceived distress severity of the somatic symptoms experienced, forgoing the need to look out for the presence or absence of a medical explanation [10]. Whether medically explained or not, the somatic symptom is significantly positively correlated with impaired physical health, psychopathology and increased healthcare use across cross-sectional clinical studies [11] and population-based surveys [12,13]. Substantial evidence points to its correlation with internalizing psychopathologies, such as anxiety, depression, post-traumatic stress, somatic anxiety [14], sexual abuse [15] and alexithymia [16]. Psycho-social factors [17], especially poverty and socio-political trauma [18], also engender somatisation among adolescents.

### 2.2. Somatisation and Poverty in Adolescence

Adolescents growing up in poverty face long-standing negative psychophysiological consequences [19,20], including psychiatric disorders [21] and stress-related somatic symptoms [22,23]. Poverty itself has been identified by researchers to be considered a significant adverse childhood experience reinforcing multiple stress-causing pathways and ultimately affecting health and well-being [24]. The absence of protective factors in such conditions can lead to emotional disorders and cognitive deficits [25]. Multiple studies have related lower socio-economic levels to an increased prevalence of somatisation within one country [26], but the same is not observed in multi-country surveys [27]. The existing correlation between financial distress and somatisation is perhaps not simply due to the lack of monetary resources but the restrictions that come along with limited monetary resources, one’s relative economic position in own society [28] and the value attached to one’s own income with respect to the country’s average [29]. Relative poverty has been commonly defined in research as an income level below the 50% median household income [30,31]. This study focused on adolescents in the lowest 25% of the median household income of each country. The relative poverty and the capability restrictions imposed because of that determine the poverty level experienced [32,33]. These restrictions can be functional, material, emotional or social in nature; the more the felt experience of being deprived or constricted because of one’s economic position in society, the more distress. Thus, an individual’s perception of poverty-led circumstances, irrespective of the economic position in society, is an important contributor to the effect of poverty. Additionally, the relationship is not a straightforward one, as social positioning and relationships with family, friends, or associates mediate the power of economic resources [34]. The negative consequences of one’s financial limitation can even be overturned by being part of a strong, supportive social network [35]. Therefore, not only relative poverty itself but also its subjective consequences are expected to facilitate a somatisation tendency. On the other hand, psychosocial protective factors that increase coping resources at disposal may dilute the negative subjective consequences of relative poverty, thus minimising the occurrence of somatisation.

### 2.3. Somatisation and Potency in Adolescence

The majority of the adolescents’ common health complaints, such as dizziness, headache and fatigue, are found to be ‘physical functional complaints’ without any direct biomedical aetiology [36], suggesting underlying emotional and behavioural difficulties, conduct disorder, depression and anxiety problem, hyperactivity/inattention and peer problems [37]. Individual differences in somatisation under similar stressors can be explained through the mediating roles of resilience [38], personal mastery, psychological well-being [39], self-esteem and perceived emotional support [40]. There is extensive work on resilience as a dynamic, complex adaptive system in the face of disruption and trauma aiding well-being [41,42,43]. In contrast, psychological resource potency is comparatively less researched. Potency was first conceived by Ben-Sira as a health-protective factor, comprising two intrapersonal (presence of mastery and confidence) and two interpersonal components (absence of alienation and anomie) [44]. It can be simply understood as one’s own confidence in self and one’s own environment to be impartial and equitable [45]. Its high presence ensures higher emotional stability and protection from the adverse effects of occasional failures in coping and resource inadequacy. Potency is known to restore emotional homeostasis disrupted during stress by weakening the link between inadequate coping and consequential adverse effects [44,45]. Previous research on potency has shown its positive role in trauma recovery and adaptation [45,46,47]. Individuals reporting somatic symptoms are often found to be using inadequate coping mechanisms [48], disturbing their emotional and bodily homeostasis and generating health-debilitating consequences [49]. Mastery or feeling self-efficacious in manipulating one’s own course of goal-directed action and confidence in one’s own capacity to overcome the demands of life are the central intrapersonal resources of adolescents [2]. They help in building a sense of strong personal agency [50], aiding in healthier coping with health-related stressors [51]. Alienation reflects powerlessness due to the inability to elicit meaningful social rewards despite their effort, expressed through social isolation, normlessness, meaninglessness and mistrust in society [52]. The psychologised concept of anomie is an individual’s feeling of disgust, anxiety and insecurity with respect to one’s social positioning, leading to pessimistic worldviews and feelings of lowered control over one’s situation [53]. In short, an absence of both alienation and anomie ensures the presence of meaningful and predictable social support. Thus, potency consisting of four factors, such as belief in a impartial and ordered society, social support, a sense of personal mastery and confidence, can be expected to prevent the tendency to somatise among the economically vulnerable. One way it perhaps does that is by strengthening the stress-coping pathway and mediating through the probable distress symptoms caused due to poverty-imposed restrictions.

### 2.4. Context of India and Israel

Poverty impositions faced by the low-income group youth in the welfare state, OECD member country, Israel, is not identical to those in the low-middle-income group country, India. The mean annual income by international standards for Israel is USD 13,271 and USD 1314 for India. GNI per capita at the PPP standard is USD 35,170 in Israel and USD 5400 in India, with 5.1% and 8.4% of income share held by the lowest 20% of the population of Israel and India, respectively [54,55,56,57]. Israel being a welfare state, the standard of living and access to material means at the disposal of their nationals is undoubtedly very different to that of Indians in similar relative poverty. Despite this sharp contrast, the implications in their capability space can be comparable when they grow up in similar relative poverty. Both countries have distinct similarities in their sociocultural blend when looking beyond the obvious differences in economic standard, size, population and literacy rates. India and Israel both have been democracies since their independence from British colonial rule in 1947 and 1948, respectively. There is political instability and a strong presence of one majoritarian religion (Hinduism in India and Judaism in Israel) while accommodating multiple ethnicities and religions [58]. Kolkata (India) and Tel-Aviv (Israel), two metropolitan cities with diverse multi-ethno-cultural flavours were selected for the study. Both are home to a considerable proportion of the urban poor. A total of 31.6% of Kolkata’s population are slum dwellers, with 7.6% economically impoverished [59] and 8.8% of the households live below-poverty-line in Tel-Aviv [60]. A total of 76.51% of Hindus make up the religious ethnicity in Kolkata [61], while Tel-Aviv has 90.8% Jews [60]. A comparative outlook of the quality-of-life and economy index surveyed by the World Bank for cross-country comparison shows similar rankings in ‘economic and political stability’ and ‘safety’ for India and Israel, while Israel has notably better civil rights and health with a lower cost of living and corruption index than India [62]. The global index of ‘safety’, as calculated by the World Bank, considers the Global Peace Index, general crime data and ongoing wars [62]. Distinct to Israel’s political situation is, however, a chronically continuing unrest in Palestine that exposes the civilian population in general to frequent armed conflicts or war. This has been cited as a key reason in the research literature explaining the high prevalence of lifetime post-traumatic disorders in the general Israeli population [63]. On the other hand, due to the geographical extensity of India, civilians in the city of Kolkata are distant from direct exposure to armed conflicts that may eventuate in the peripheral zones of the country from time to time. Overall, the sociocultural, political and economic context of both cities generates precursors to somatisation tendency [17,18]. The gross indicators put Israel socio-economically in a more advantageous position over India while having a comparable ethno-cultural blend. Comparing the potency and ST in low-income group youth from these two countries is expected to yield insight into the relative poverty–somatisation–potency relationship.

### 2.5. Aim of the Study

**Figure 2:**
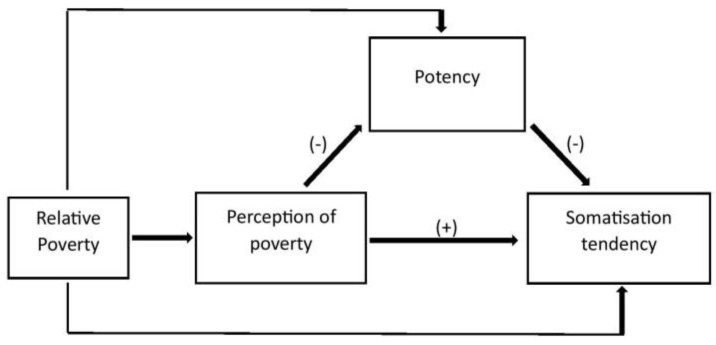
Conceived relationship between potency, relative poverty and perception of poverty with somatisation tendency. The signs denote the direction of relationship between variables.

Research on urban youths highlights somatic complaints to be one of the most sensitive indicators of distress [64], coupling as an important warning sign and intervention ground to prevent ongoing emotional and behavioural problems. Social inequality and relative poverty have also been found to precipitate negative health consequences regardless of one’s absolute economic level [65]. The theoretical background indicates that relative poverty and felt experience of poverty can contribute to ST, and the presence of psychosocial protective factors such as potency are possible mediators of this relationship. Figure 2 illustrates this conceived relationship. The figure indicates relative poverty as a common trigger to adverse experiences affecting the potency and perception of poverty of adolescents in relative poverty. It is further conceived that perception of poverty or the felt experience of relative poverty impacts ST via the mediating variable potency, which independently also has been theoretically deduced to impact ST. The present study attempts to contribute to the limited pool of studies on youth living in relative poverty and their bodily consequences of psychosocial conditions [18]. It specifically explores how potency is related to ST in the relatively poor adolescents (12–16 years) of a low-middle-income group country (India) and a high-income group country (Israel). The present study has three aims: (i) To explore the nature of ST and potency among urban community adolescents from India and Israel living in relative poverty; (ii) to understand the relationship between potency, PP and ST among the studied group and (iii) understand the mediating role of potency in the relationship between PP and ST.

## 3. Materials and Methods

### 3.1. Participants

Income level, determining the relative poverty of the participants in comparison to their own country’s average [66], was used as the prime inclusion criteria for this study. Those in the lowest quartile (25%) of the country’s income in purchasing power parity (PPP) measure were defined as the low-income group for this study. A method of converting the wages in the individual country’s currency to PPP value on a global scale, as published by BBC [67], was used here to ascertain the per capita income level in Indian and Israeli national currencies. The PPP is used to compare levels of poverty across countries [68] and generally to equalise the purchasing power of different currencies by eliminating the differences in price levels between countries [69]. Purposive sampling was used to access the concerned youth population (12–16 years). Permission was sought from educational institutions and academic bodies in Kolkata and Tel-Aviv for the study and access to the socio-economic details of their students. Only those meeting the inclusion criteria were approached for consent to be part of the study. The adolescents from India (N = 200) lived in slum neighbourhoods of central and north Kolkata, attending free government primary schools and informal non-governmental schools, the only accessible educational institutions to the economically challenged in India. Many of them were first-generation learners and engaged in unstructured labour, helping in their parents’ odd jobs (e.g., collecting rags and selling farm produce). Very few were engaged in part-time work. Adolescents from Israel (N = 208) belonged to low-economic settlements in the southeast and southern parts of Tel-Aviv, attending public schools, as identified by the Israeli Ministry of Education to match the inclusion criteria. The Israeli youth, some of whom themselves were engaged in part-time work besides being students, had mostly educated, working parents. The contrast in the parental educational level and nature of occupation in the lowest income quartile population is perhaps a reflection of the difference between a developed and a developing economy. The sociodemographic details of the participants are given in Table 1.

### 3.2. Procedure

As per research protocol and ethical committee guidelines from the respective country’s authorities, similarity in the procedural phase of the data collection was adhered to as much as possible. The research proposal was approved by the Committee of Graduate Studies Authority from the University of Haifa, and three research associates from there collected the data from Israel as part of an ethically approved dissertation (approval No.: 123/15) under the guidance of one of the authors. The investigating researcher collected data in India. Permission for administering the research tools there was obtained from the Institutional Ethics Committee, University of Kolkata, India. Individual assent form following the WHO’s guidelines for adolescent research was used in this study. Adolescents with diagnosed organic or psychiatric conditions ascertained by self-report were excluded from the study. An interview schedule designed to record the sociodemographic details for this study was used first, followed by the questionnaires measuring ST and potency. A rating scale measuring perception of poverty (PP) was developed for this study and was used with Indian adolescents. The Israeli Ministry of Education restricted the use of this measure with Israeli adolescents. They reported their perceived level of household income in comparison to what they think to be the average Israeli household earning as much or slightly below and much or slightly above the average of their country. However, this verbal report was not considered as a measure for understanding their perception of poverty in this study. All questionnaires used for the study were translated into the regional languages—Bengali (for Kolkata), Hebrew and Arab (for Tel-Aviv). The translation–back–translation method [70,71] was followed aiming at preserving the same ideas across linguistic boundaries [72]. The complete data collection and accumulation process was completed from May 2015–October 2017.

### 3.3. Measurement Scales

Somatisation tendency: Bradford Somatisation Inventory (BSI-21) developed by Mumford et al. (1991) was used to measure ST, having a cut-off of 13/14 for patients seeking medical help, with sensitivity and specificity against the non-psychiatric population of 0.87 and 0.75, respectively [73]. The response category of the original scale has three options: absent, present on less than 15 days during the past month and present on more than 15 days during the past month. BSI-21 has been used among both community and clinical samples across ages, including young student populations, cross-nationally. Cronbach’s alpha calculated for the translated BSI-21 were 0.78 (Bengali), 0.88 (Hebrew) and 0.91 (Arabic). This study used only the total somatic symptom count found to provide a ready indicator of the health status in epidemiological studies [12]. Therefore, the presence of a symptom within the past month was scored as 1 and no score was allotted to the absence of symptoms. The total attainable range of score was 0–21.

Potency: The Potency Scale [44] was used, having 19 items measuring 4 dimensions: presence of mastery (6 items), presence of self-confidence (3 items), absence of alienation (5 items) and absence of anomie (5 items). All items were measured on a scale of 1–6, where 1 indicates “totally agree” and 6 indicates “totally disagree”. The Potency Scale has been found to be a reliable and valid measure in research studies across different ethnic groups in Israel [44] and India [46]. Individual dimension score, as well as a total score, is available for potency. Cronbach’s alpha calculated for the translated Potency Scale was 0.83 (Bengali), 0.88 (Hebrew) and 0.91 (Arabic).

Perception of poverty: The perceived impact of the physical and emotional experiences of poverty was defined as the perception of poverty for this study. A short 15-item rating scale survey questionnaire was developed for this study due to the paucity of quantifiable scales measuring adolescents’ perception of their level of poverty. The five-point Likert-type numerical rating scale was conceived as an index based on a relevant, limited set of conceptually associated items. The rating scale items were framed based on the interviewed responses in a qualitative study by Halik and Webley (2011) enquiring low-income group Malaysian adolescents about their personal perception of poverty [74]. The content validity of the scale items was determined by the experts’ rating (N = 2) in the dimensions of relevance, representativeness and clarity [75,76]. The decision to retain an item was taken when both the experts agreed upon its representativeness and relevance of the construct measured. The items were modified according to the expert’s judgment in the clarity of items and critiqued on the structure and content of the item [77]. Each item had a response range option from ‘almost always’ (scored as five) to ‘never’ (scored as one). Seven of the items pertained to the feeling associated with the state of experienced poverty (such as, “I feel helpless because of my present economic condition”), and eight of the items pertained to how the adolescents see the functional effect of poverty in their own life (such as, “I think if there was more money in my family, I would have been healthier”). One item among the fifteen was reverse scored. The total score derived from this scale gave an index of subjective poverty. A high score on the scale was indicative of a more negative attribution of poverty’s impact on one’s own life. Cronbach’s alpha calculated for the translated scale was 0.819 (Bengali).

### 3.4. Data Analysis

IBM SPSS 20 statistical software was used for statistical computations. Descriptive statistics were computed to understand the nature of ST, the level of potency and its dimensions. Non-parametric correlational and inferential statistics were used as the research variables did not follow normality in the participant population (significant results, *p* < 0.05, were found in the K-S Test of normality). Cross-country difference between the nature of somatic symptom reports was seen using the Chi-square test of difference. The Mann–Whitney U test was computed to explore sex and between-country differences. Spearman rank correlation and multiple linear regression analysis assessed the degree and predictability of the relationship between the research variables. Three parameters of the multiple linear regression are reported [78]: the unstandardized regression coefficient (b), standardized regression coefficient (β) and adjusted R^2^. Percentage change in estimate of ‘b’ before and after adjusting the sociodemographic variables was checked for any confounding effect [79] between potency and ST. A change in rate ratio estimate of 10% or beyond is considered significant, requiring controlling the variable with a confounding effect [80]. Percentage change after adjusting for age, sex, education, religion, the participants’ occupation, family size, parental education and employment status were insignificant for both countries. Finally, the independent variables were entered stepwise to assess the relative contribution of each of the dimensions of potency in ST. The variance inflation factor (VIF) for each of the variables included in the model is reported to keep a check on the possible multicollinearity present within the dependent variables themselves. A VIF value from 1 to 4 is deemed acceptable [81]. Additionally, for the Indian participants, the conceived mediating effect of potency on the relationship between PP and ST was tested with simple mediation analysis using PROCESS [82]. The outcome variable for analysis was ST, the predictor variable was PP, and the mediator variable was potency. A series of linear regression was used to determine the mediation, expressed with three paths of regression analysis—path ‘a’ denotes the predictive relationship of the predictor on the mediator variable, path ‘b’ denotes the predictive relationship of the mediator on the outcome variable and path ‘c’ denotes the predictive relationship of the predictor on outcome variable in the presence of the mediator. The statistically significant indirect effect (path a × b), along with the absence of zero between the confidence intervals, was considered proof of the mediation effect [82,83].

## 4. Results

### 4.1. Nature of ST, Potency and PP

#### 4.1.1. India

**Figure 3:**
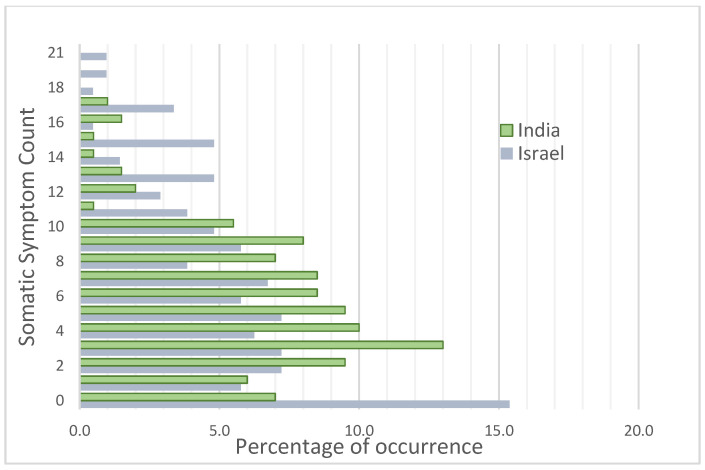
Distribution of ST in the participant population: India - Israel Comparison.

Table 2 shows that Indian adolescents reported an average of 5–6 somatic symptoms. A total of 1% of Indian youth simultaneously experience a maximum of 17 symptoms, and 7% are symptom-free (Figure 3). Table 3 shows that lack of energy and headaches are most experienced by the Indian low-income youth, followed closely by tightness in the neck and shoulders and feeling tired even when not working, while the least-reported symptoms were of sinking heart and frequent urination. Table 4A shows that Indian females report an overall significantly higher ST over males (U = 3827.50, Z = −2.66, *p* < 0.001). Females perceive a lower sense of mastery (U = 3970.5, Z = −2.30, *p* ≤ 0.05) and a higher sense of alienation (U = 4105, Z = −1.97, *p* ≤ 0.05) and anomie (U = 3987.5, Z = −2.252, *p* ≤ 0.05) than males but do not differ in confidence to a significant degree (*p* = 0.340). Higher PP (U = 4019.5, Z = −2.179, *p* ≤ 0.05) and lower potency (U = 3990.0, Z = −2.25, *p* ≤ 0.05) in females than males theoretically corroborates with the obtained higher presence of ST among Indian females than males.

#### 4.1.2. Israel

Israeli adolescents report an average of 6–7 somatic symptoms. All 21 somatic symptoms are experienced simultaneously by 1% of Israeli youth, and 15.4% of them are completely symptom-free (Figure 3, explaining the high variance in the data. Table 3 indicates that feeling tired, headache, weakness, fluttering in the stomach and tension in the head and shoulders are experienced by over 40% of the Israeli low-income youth. In comparison, less than 20% of the participants report frequent urination and constipation. Table 4A shows weak evidence of females reporting higher symptom frequency than males (U = 4547, Z = −1.94, *p* = 0.053).

#### 4.1.3. Cross-Country Comparison

Table 2 shows the mean (SD) of ST among Indian and Israeli adolescents were 5.5 (3.7) and 6.6 (5.4), respectively, and Table 4B show no major evidence of a difference in ST reported by the adolescents from both countries [U = 19,217.1, Z = −1.33, *p* = 0.182]. Table 3 reveals a commonality in somatic symptoms reported in general, with around 50% complaining of headaches. Pain symptoms such as headache, pain in the neck and shoulder and general weakness are the most reported somatic complaints among all, while frequent urination is one of the least reported symptoms. Israeli adolescents reported a higher frequency of symptoms, such as dry mouth, pressure on the chest or inside head, etc., than Indian counterparts (*p* < 0.05). The Indian adolescents are higher than the Israeli counterparts in feelings of weakness or lack of energy (*p* < 0.05). Table 4B shows that Indian adolescents have an overall higher presence of potency than their Israeli peers (U = 1385.5, Z = −16.31, *p* < 0.001). A similar result is seen for all three potency dimensions except anomie. The same trend is reflected between both males (U = 162.5, Z = −11.21, *p* < 0.001) and females (U = 571.5, Z = −11.79, *p* < 0.001).

**Table 2:** A comparative overview of descriptives of the research variables across India (Kolkata) and Israel (Tel-Aviv).

Variables	India Mean (SD)			Israel Mean (SD)		
	Total	Male	Female	Total	Male	Female
Somatisation Tendency	5.5 (3.7)	4.7 (3.5)	6.1 (3.8)	6.6 (5.4)	5.9 (5.3)	7.2 (8.2)
Potency	127.5 (31.7)	133.6 (30)	122.9 (32.4)	64.0 (14.5)	63.6 (14.6)	64.4 (14.4)
Mastery	76.5 (20.1)	80.33 (18.9)	73.5 (20.6)	20.4 (5.5)	20.1(5.6)	20.6 (5.3)
Self-confidence	13 (4.5)	13.3 (4.6)	12.8 (4.5)	10.9 (3.1)	10.6 (3)	11.1 (3.1)
Alienation	19.5 (6.6)	20.5 (6.5)	18.7 (6.6)	14 (4.6)	13.9 (4.4)	14 (4.6)
Anomie	18.6 (5.5)	19.5 (5.3)	17.9 (5.5)	18.8 (5.6)	19 (5.7)	18.7 (5.5)
Perception of Poverty	34.8 (10.75)	32.9 (10.4)	36.2 (10.8)	-	-	-

**Table 3:** A comparative frequency of the nature of symptom presence between Indian and Israeli adolescents.

Sr. No.	Symptoms According to BSI-21	% Reporting Presence		χ2
		India	Israel	
1	Severe headaches	52	50	0.02
2	Fluttering or a feeling of something moving in the stomach	33	42	4.35 *
3	Pain or tension in the neck and shoulders	41	43	0.31
4	Feeling constriction of the head	20	28	4.88 *
5	Pain in the chest or heart	27	33	2.57
6	Dry mouth or throat	16	33	16.53 ***
7	Lack of energy (weakness)	59	41	12.14 ***
8	Excessive sweating	12	33	26.85 ***
9	Pressure or tightness in the chest or heart	11	23	10.91 ***
10	Choking sensation in the throat	14	23	5.35 *
11	Body aches or pains	43	35	2.64
12	Palpitations (heart pounding)	29	27	0.11
13	Trembling or shaking	32	25	2.72
14	Passing urine more frequently	6	18	13.68 ***
15	Head felt heavy	21	32	6.96 **
16	Feeling tired, even when not working?	41	42	0.12
17	Feeling pressure inside the head	18	38	22.4 ***
18	Constipation	15	15	0.02
19	Heart feels weak or sinking	7	20	15.14 ***
20	Excessive wind (gas) or belching	33	24	4.37 *
21	Hands or feet feel cold	24	33	3.91 *

* *p* ≤ 0.05, ** *p* ≤ 0.01, *** *p* ≤ 0.001.

**Table 4:** (**A**) Mann–Whitney U test showing differences between males and females in ST, PP and potency. (**B**) Mann–Whitney U Test showing differences in the potency and ST between Indian and Israeli adolescents, Indian and Israeli males and Indian and Israeli females.

**(A)**											
**Component**	**Sex**		**Mean Rank India**		**U**	**Z**	**Mean Rank Israel**		**U**	**Z**	
Somatisation tendency	Male		88.01		3827.50 **	−2.66	95.88		4547.00	−1.94	
	Female		109.93				112.04				
Potency total	Male		111.10		3990.00 *	−2.25	101.09		5052.50	−0.77	
	Female		92.50				107.48				
Mastery	Male		111.33		3970.50 *	−2.30	102.78		5216.50	−0.39	
	Female		92.33				106.00				
Confidence	Male		104.95		4519.50	−0.95	98.78		4828.50	−1.29	
	Female		97.14				109.50				
Alienation	Male		109.77		4105.00 *	−1.970	102.62		5201.00	−0.42	
	Female		93.51				106.14				
Anomie	Male		111.13		3987.50 *	−2.262	104.19		5353.50	−0.07	
	Female		92.48				104.77				
Perception of poverty	Male		90.24		4019.50 *	−2.179	--		--	--	
	Female		108.24								
(**B**)											
**Component**	**Country**	**Mean Rank Total**	**U**	**Z**	**Mean Rank Male**		**U**	**Z**	**Mean Rank Female**	**U**	**Z**
Somatisation tendency	India	196.59	19,217.00	−1.33	89.11		3922.50	−0.70	107.93	5749.50	−1.19
	Israel	212.11			94.56				118.20		
Potency total	India	301.57	1385.50 ***	−16.3	138.61		162.50 ***	−11.21	163.49	571.50 ***	−11.79
	Israel	111.16			50.68				61.15		
Mastery	India	307.33	235.00 ***	−17.3	140.16		29.50 ***	−11.59	167.68	93.00 ***	−12.77
	Israel	105.63			49.30				56.84		
Confidence	India	236.96	14,308.00 ***	−5.48	110.00		2623.00 ***	−4.35	127.44	4680.50 ***	−3.39
	Israel	173.29			76.04				98.17		
Alienation	India	253.90	10,920.00 ***	−8.31	120.22		1744.50 ***	−6.79	134.58	3867.00 ***	−5.05
	Israel	157.00			66.98				90.84		
Anomie	India	201.37	20,174.50	−0.526	95.94		3832.00	−0.95	107.03	5646.00	−1.4
	Israel	207.51			88.51				119.14		

* *p* ≤ 0.05, ** *p* ≤ 0.01 and *** *p* ≤ 0.001.

### 4.2. Relationship between ST, Potency and PP

#### 4.2.1. India

All three variables have a significant correlation (*p* < 0.001) between themselves. Table 5 shows that PP and ST are expectedly positively correlated (*p* = 0.541), and potency is negatively correlated to both PP (*p* = −0.598) and ST (*p* = −0.376).

The regression model R1, with the dimensions of potency as the independent variable and ST as the dependent variable (Table 6A, includes mastery, alienation and confidence, each affecting the dependent variable in significant proportions amounting to 38.8% of its variance [R^2^ = 0.397, F(1, 196) = 43.008, *p* < 0.001] excluding out anomie. A hierarchical regression to see the predictability of PP and potency on ST is also obtained, PP is entered in Step 1, and potency is added in Step 2. Model R1.1 show that when potency is controlled, PP alone predicts 30.2% of the variance in ST [R^2^ = 0.306, F(1, 198) = 87.21, *p* < 0.001]. On potency being added to the model, a significant R^2^ change (ΔR^2^ = 0.122, *p* < 0.001) is observed, and the unstandardized regression coefficient (b) for PP is reduced from 0.192 to 0.103. The final model of R1.1 (Step 2) predicts 42.2% of variance in ST [R^2^ = 0.428, F(2, 197) = 73.74, *p* < 0.001].

#### 4.2.2. Israel

Table 5 shows potency, and all its dimensions are significantly negatively correlated with somatisation tendency (*p* < 0.05). Potency emerges as a significant predictor of ST, with anomie and mastery having the highest R^2^ values (0.1024 and 0.1018, respectively). Table 6B show mastery and anomie produce 13.2% of the variance in the ST score for Israel [R^2^ = 0.141, F(1, 205) = 16.786, *p* ≤0.001], excluding confidence and alienation.

#### 4.2.3. Cross-Country Comparison

Table 6 show that potency emerges as a stronger predictor of ST among Indian adolescents than in Israel. The final predictive model for Indian adolescents includes both the intrapersonal resources (mastery and confidence) and one interpersonal resource (alienation) of potency, and that for Israeli adolescents, it includes one intrapersonal (mastery) and one interpersonal resource (anomie) of potency. The common significant predictor for both Indian and Israeli adolescents’ ST is mastery (b = −0.047, *p* = 0.003, and b = −0.216, *p* = 0.003, respectively). Confidence and alienation emerge as significant contributors only for Indian adolescents (*p* = −0.152, *p* = 0.017 and b = −0.167, *p* < 0.001, respectively). While anomie emerges as the stronger predictor for Israeli adolescents (b = −0.212, *p* = 0.003).

**Table 5:** Spearman’s Correlation between ST, Potency, and PP.

Variables	Country	Somatisation Tendency	P	M	C	Al	A
Potency Total (P)	India	−0.376 ***					
	Israel	−0.608 ***					
Mastery (M)	India	−0.354 ***	0.740 ***				
	Israel	−0.603 ***	0.985 ***				
Confidence (C)	India	−0.312 ***	0.705 ***	0.471 ***			
	Israel	−0.511 ***	0.747 ***	0.716 ***			
Alienation (Al)	India	−0.170 *	0.685 ***	0.346 ***	0.362 ***		
	Israel	−0.537 ***	0.754 ***	0.681 ***	0.502 ***		
Anomie (A)	India	−0.278 ***	0.790 ***	0.393 ***	0.458 ***	0.439 ***	
	Israel	−0.390 ***	0.722 ***	0.662 ***	0.448 ***	0.489 ***	
Perception of Poverty	India	−0.541 ***	−0.598 ***	−0.567 ***	−0.431 ***	−0.535 ***	−0.500 ***

* *p* ≤ 0.05and *** *p* ≤ 0.001.

**Table 6:** (**A**) Multiple linear regression, India, R1—IV: potency dimensions and DV: ST. R1.1—IV: PP and potency and DV: ST. (**B**) Multiple linear regression, Israel, R1—IV: potency dimensions and DV: ST.

**(A)**												
**Model**	**Predictors**	**Unstandardized Coefficients**		**Standardized Coefficient**	**Sig. Level**	**R**	**R sq.**	**Adj. R sq.**	**SE**	**R Sq. Change**	**F**	**Sig. Level**
		**b**	**Std. Error**	**β**								
R1	(Constant)	14.335	0.832		<0.001	0.63	0.397	0.388	2.928	0.018	43.008 (3, 196)	<0.001
	Mastery	−0.047	0.016	−0.254	0.003							
	Alienation	−0.167	0.04	−0.295	<0.001							
	Confidence	−0.152	0.063	−0.184	0.017							
R1.1 Step 1	(Constant)	−1.204	0.751			0.553	0.306	0.302	3.125	0.306	87.217	<0.001
	Perception of poverty	0.192	0.021	0.553								
R1.1 Step 2	(Constant)	8.443	1.635		8.443	0.654	0.428	0.422	2.844	0.122	73.740	<0.001
	Perception of poverty	0.103	0.023	0.296	0.103							
	Potency	−0.051	0.008	−0.434	−0.051							
**(B)**												
**Model**	**Predictors**	**Unstandardized Coefficients**		**Standardized Coefficient**	**Sig. Level**	**R**	**R sq.**	**Adj. R sq.**	**SE**	**R sq. Change**	**F**	**Sig. Level**
		***B***	**Std. Error**	**β**								
R2	(Constant)	14.989	1.495		<0.001	0.375	0.141	0.132	5.004	0.038	16.786 (2, 205)	<0.001
	Anomie	−0.212	0.069	−0.221	0.003							
	Mastery	−0.216	0.072	−0.219	0.003							

### 4.3. Potency as a Mediator Variable

#### India

**Figure 4:**
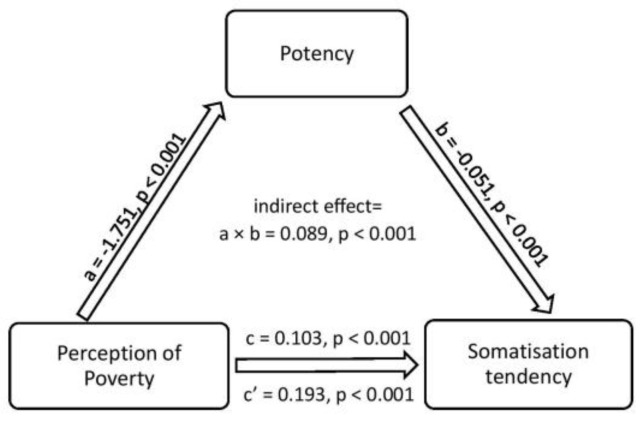
Schematic representation of the mediation analysis with potency as the mediating variable, perception of poverty as the predictor variable and somatisation tendency as the outcome variable.

The mediational effect of potency on the relationship between PP and ST is obtained with simple mediation analysis using PROCESS. It reveals a significant indirect effect (path a×b) of the impact of PP on ST (b = 0.09, 95% C.I. = 0.058, 0.125), supporting the hypothesized relationship in this study. The path ‘a’ or relationship between PP and potency is found to be negative and significant (b = −1.75, *p* < 0.001), indicating that the higher the PP, the more likely the potency will be lower. The path ‘b’ or relationship between potency and ST is found to be negative and significant (b = −0.05, *p* < 0.001), indicating that the more the potency, the less ST will be. Additionally, the direct effect of PP on ST in the presence of the mediator (path c) is found to be positive and significant (b = 0.103, *p* < 0.001), indicating that the higher the PP, the ST will also tend to be higher. Therefore, potency partially mediates the relationship between PP and ST. The summary with total, direct and indirect effects is presented in Table 7, and Figure 4 shows the schematic representation of the path relationship.

## 5. Discussion

The somatisation tendency was found to be present to a clinically significant degree in low-income group community youths from both India and Israel. As conceptualized, potency significantly negatively correlated and predicted ST and the obtained results for Indian participants show that potency partially mediates the effect of the perception of poverty on ST. In other words, the perception of poverty of Indian youth in relative poverty impacts somatisation tendency through a mediating effect of potency. Strong evidence for females having higher ST and corresponding lower potency than males was obtained only for Indian participants.

### 5.1. Nature of Somatic Symptoms Reported—Cross-National Perspective

Both the transitional age of adolescence and the relative poverty may have been instrumental to the high prevalence of ST in youth [84] across nations. There was no strong evidence that the youth from a low-middle income group country, India, experienced higher ST than their peers in the high-income group country—Israel. The youth in relative poverty reported 5–7 somatic symptoms on average. Because three or more somatic symptoms, irrespective of identified organic cause, predict psychopathology, substance use and future medical service use [85], both Indian and Israeli community adolescents can be recognized to be at risk. A total of 15.4% of Israeli youth and 7% of Indian youth were also found to be devoid of any somatic symptoms. This can be because of developing emotional habituation under continuous threat [86] that buffers one to stress, lowering psychophysical manifestations of the same [87]. The comparatively lesser proportion of symptom-free adolescents in India may reflect the sociocultural influence that reinforces somatic expression of psychological distress more than in Western societies [88]. Somatic symptoms have also been very commonly associated with both personal life trauma and non-personal, such as politically motivated situations of terrorism and war [89] or social deprivation and exclusion in underprivileged sections of society [90]. The latter can be deemed for the studied youth in both Israel and India, while trauma and anxiety disorders are reported commonly among Israeli youth due to continued exposure to political conflicts [91] and war-like situations [92]. Focusing on the nature of common somatic symptoms reported cross-nationally, headaches were the most common symptom, followed by feeling a lack of energy, tired when not working, pain in the neck/shoulders and having a fluttering feeling inside the stomach. Self-reported cardiovascular symptoms [93] were significantly more common in Israeli adolescents (e.g., pressure in the chest, sinking or weak heart) in contrast to musculoskeletal pain symptoms such as headache, pressure, or tightness on shoulders and lack of energy for the Indians. Socially threatening events, such as social rejection [94] and armed conflicts, whether in physical proximity or not [86], arouse trauma and can result in sympathetic activation leading to cardiovascular symptoms such as racing/pounding heart [95], shortness of breath or chest pain [96], tension headache [97] and pain symptoms [98]. Heightened weakness reported by Indian adolescents may suggest their comparatively poorer living conditions and nutritional deficiency in comparison to the Israeli youth [55]. There is a need to understand the common and/or unique psychosocial contributors to different somatic-symptom clusters [99] before concluding. However, the similar level of ST in low-income group youth, irrespective of the national economic status, reinstates the significance of relative poverty in adolescent mental health over absolute poverty [100,101].

### 5.2. Potency—Somatisation Tendency—Cross-National Perspective

Potency as a resource has not been experimentally assessed in the context of ST before. However, multiple studies have independently highlighted that social isolation and exclusion [102], social support [101], social cohesion and control [103] and mastery [104] mediate the role of economic class and mental health, especially somatisation and mood disorders. The composite resource of potency wraps in similar protective factors together—the presence of mastery and confidence on one side and the absence of alienation and anomie on the other. Mastery and confidence provide a sense of self-capability and belief in oneself to enact upon the environment. Social alienation and anomie lead to feelings of being excluded from the larger society, having no control over or predictability of one’s own society and society being unjust [46]. Therefore, strong evidence of potency (and all its dimensions) negative correlation with ST not only dovetails previous findings but also helps to focus on the specific combination factors affecting ST. It also emerged as the common significant predictor of ST, and on assessing the role of the individual dimensions, a variation was observed in their relative importance to each country. Potency, as a composite factor, explained 13.2% and 38.8% of the variance in the score of ST in Israeli and Indian adolescents, respectively. The mastery dimension emerged as the only common significant contributor to ST cross-nationally. The period of adolescence poses a challenge in exercising self-control both over self and external situations, escalating its need felt more than ever. Thus, the presence of mastery serves as a protector against felt distress, mediating the stress–pathology pathway [105], enabling effective dealing with forthcoming stressors [106,107], and its depletion associated with somatisation in low-income urban youth [108]. The presence of confidence emerged as a strong predictor only for Indian adolescents. Confidence enables one to mobilize required resources as per need, contributing to mastery attainment. With poorer living conditions than in Israel [62], Indian adolescents are likely to require higher skills in mobilizing resources to sustain a minimum living standard. Thus, for the Indian youth, the presence of confidence, independent of mastery, may have facilitated sociability and resource manipulation [109], mediating the distress–pathology pathway. Among the interpersonal resources, anomie was the strongest independent predictor of ST for Israeli adolescents, while alienation was significant in the Indian context. It is also important to note here that both Indian and Israeli adolescents reported a similar level of anomie in their respective societies (Table 4B. It is experienced when one’s effort to attain legitimate goals is thwarted due to the inaccessibility of the legitimate resources required to attain the goal. One of the many adverse consequences of anomie is rejecting both pursuits of goal and the present social norms, leading to drug abuse, mental illness and escape [110]. According to the obtained results, Indian and Israeli youth living in relative poverty bears similar notion about the situation of distributive order in their own societies, but one (Indian) is probably more tolerant and adjusting to that; while the other’s expectations clash with its given state, making it a significant contributing factor to somatic distress. Indian society is more accepting of the unequal power distribution, uncertainty, and ambiguity within it than Israel [111,112]. Habitual conditioning has probably led the low-income group of Indian youth to ‘normalize’ their inaccessibility of legitimate resources. Thus, when other dimensions were controlled for, anomie was independently not consequential in the Indian adolescents’ somatisation tendency. High intolerance to power disequilibrium in accessing common social resources and ambiguity in distributive social justice characterizes the Israeli population as much as they value modesty, societal cooperation, caring for the weak and quality of life [112,113]. Such a constellation of cultural value indicators may have elevated the significance of anomie to Israeli adolescents. The studied Israeli participants comprised 63.5% Arabic nationals, who experience more socio-politically triggered anomie than Jewish nationals [114,115]. This may have as well contributed to the overall sense of anomie reported by the low-income group of Israeli youths. The other dimension—alienation—was perceived higher in Israeli youth but emerged as a stronger predictor of ST for only the Indians. The sense of alienation develops from a sense of non-belongingness and disconnect with one’s own immediate society [116], arousing hopelessness. Economic class mediates social cohesiveness, where people in poverty face more alienation and invisibility [117,118] and lack of proper educational and medical infrastructural facilities [119]. Restricted inter-class mobility within Indian societies [120] compelling the youth to live within a compromised socio-economic sphere over time may have made sense of alienation more sensitive with respect to ST.

### 5.3. Potency—Somatisation Tendency—Sex Difference

Females were found to report higher ST in general, but the evidence is stronger for the Indian youth (*p* ≤ 0.05). Multi-country studies have confirmed both types of findings, with the literature being heavier on the side of females being more prone to somatisation [121]. Females reporting a higher frequency of somatic symptoms, in greater intensity, have been empirically associated with a higher incidence of anxiety disorders, depression and psychiatric disorders [122] on the one hand and widespread social oppression on the other [22]. Research highlights that low-income group Indian females face greater challenges in mobilising socio-economic resources than males [123,124]. Correspondingly this study found strong evidence of lower potency in Indian females than their male peers living in the same relative poverty (*p* ≤ 0.05). Specifically, females had lower mastery, higher alienation and anomie (*p* ≤ 0.05), but the difference in ‘confidence’ with males was weak. Such differences in potency or in any of its dimensions were not evident among Israeli adolescents in this study as well as in previous research [47]. It is likely that Indian females have experienced a less accommodating and integrative society than their male peers. Capability deprivation [32] that comes along in a unique way with economic and societal restrictions for Indian females can explain their lower potency, which in turn can affect the ST. Externally imposed social constraints based on sex, irrespective of the acquired confidence, can pose a threat to goal-oriented movements lowering chances of mastery attainment. Indian females particularly face inequitable, restricted infrastructural and financial support [124] to the extent of a strong negative bias towards female education [125]. Doing familial chores, for example, is considered more important for the girls of Indian families than attending school [119,126]. The Indian females in relative poverty represent a subclass facing unique inequality associated with gender-specific bias [125] within their already alienated populace, thus becoming more prone to somatic manifestations of distress [127] and poor mental health [128].

### 5.4. Somatisation Tendency, and Perception of Poverty and Potency—A Slice from India

This study explored the role of potency in somatisation tendency against a very specific background—economic poverty, which itself has been identified as one of the causal and/or contributory factors of ST [84]. Poverty continues to be a global concern for mental and physical health status because of its widespread impact in all areas of life—from accessing clean, sanitized living conditions, medical facilities and educational and career opportunities to inter-class mobility across various societal levels [129]. The impact is not limited to the absolute economic position one holds in society but is felt continuously in the restrictions imposed by society over access to various needs, desires, and even aspirations one has. The study thus hypothesized that the felt impact of one’s economic position or the perception of own poverty be significantly related to ST and potency both. It is through potency that ST impacts low-income group adolescents. A 5-point Likert-type scale developed for this study measured the individual’s perceived opinion about the impact of their economic poverty on different areas of life, such as education, health, luck, personal desires, etc., and the summated score derived from it gave the index of ‘perception of own poverty’. Though no use of such a scale has been found in earlier studies on somatisation, previous research indicates a strong relationship between adolescents’ subjective economic status and self-rated health status, more than the objective markers of their socio-economic status [130,131]. The study results validate the theoretical position that for adolescents belonging to similar relative poverty, perception of poverty significantly predicts ST and this relationship is significantly mediated by potency, a psycho-social resource (Figure 4). Mental health is further compromised for low-income group youth with ‘unfavourable perceptions’ of their own economic position [100], but further studies are required to understand how the objective and relative economic positions interact or relate to impact the youth’s mental health. Research also puts forward the significance of lowering perceived poverty for improving mental health [101]. Though further studies are required to conclude this, the present study’s findings offer potency as a plausible explanation for this link in the context of somatisation tendency in low-income group adolescents.

## 6. Conclusions

In summary, potency comes up as a significant contributor to ST for low-income group adolescents from India and Israel. Though the nature of ST differs between the youth from the two countries, both are at high-risk for future psychopathologies, unhealthy lifestyle habits and increased medical burden. The detailed understanding of potency dimensions and their predictive capacity of ST in studied adolescents is supported by both the psychosocial [132] and sociocultural [22] causation hypotheses of somatisation. Potency is further established as the mediating variable impacting the relationship between perceived poverty and somatisation tendency in low-income community youth. Weakening subjective poverty over alleviating objective financial deprivation is an effective buffer against the negative psychological impact of poverty [101]. An in-depth scientific study on potency development can create avenues for building person- or group-specific social support networks and enabling measures that can enhance one’s sense of mastery and competence. Such protective frameworks not only can systematically prevent ST but also safeguards from impacts of adversities. The presence of informational and instrumental support and welfare schemes aiding financial capability for those in poverty can increase the sense of belongingness and trust in one’s society and enhance the lived experience positively, in turn weakening poverty’s link to poor mental health.

This study has some limitations that can be taken up for future research expanding on the finding of this study: (1) The measure of perceived poverty of adolescents was specifically developed for this study, which also served as the pilot measure for the survey scale. Previous studies either have used single generalized survey questions, such as “Compared to others, how would you rate your family’s economic situation?” [100] or in-depth semi-structured interviews [74]. While interviews in large-scale studies may not be an optimal option, a perceived ranking of own economic position compared with anonymous others can have the risk of oversimplifying or underestimating the adolescents’ understanding of their economic situation. This survey scale attempted to provide a comprehensive quantifiable measure of poverty’s perceived impact on a daily functional level and its associated emotional effect on youth. Further studies would be required to establish the validity of this measure. (2) A measure of perception of poverty could not be taken from Israeli participants, limiting the scope of the finding. (3) The potential factors impacting the poverty—ST links other than potency, such as perceived family and peer support [34,35], hope and optimism, negative life events and perceived stress, were beyond the scope of analysis in this study. The inclusion of such factors will deliver a more comprehensive model of the poverty–ST pathway. (4) The cross-sectional nature of the study and (5) wider participant representation in terms of different ethnicities could strengthen the current findings for these two multi-ethnic countries. The finding of sex-based differences in potency and ST can also be of future research interest, especially in the context of different cultures.

## Figures and Tables

**Table 1:** A comparative overview of the participant population across India (Kolkata) and Israel (Tel-Aviv).

Kolkata	Details	Tel-Aviv
14.08 (1.19)	Age: Mean (SD)	15.21 (0.77)
Male—43, Female—57	Sex (%)	Male—57, Female—43
Hindu—92.5, Muslim—1, Christian—6.5	Religion (%)	Jew—36.5, Arab—63.5
Indian—100%	Nationality	Jew—36.5%, Arab—63.5%
India	Birth Country	Israel
4.6 (1.3)	Family Size: Mean (SD)	4.7 (3.7)
Illiterate—36.2 (F), 31.3(M) Primary Education—36.7(F), 31.3(M) Middle School—19 (Both) High School—1.7(F), 2.7(M) Undergraduate Degree—1.5 (Both)	Parental Educational Level (%) Father (F), Mother (M)	Primary Education—9.1(F), 7.5(M) Middle School—12.7(F), 14.9(M) High School—57.9(F), 57.7(M) Undergraduate Degree—20.3(F),17.9(M)
Unemployed—3.9(F), 45.6(M) Part-time Workers—4.5(F), 27.5(M) Daily Labour—44.7(F), 15.5(M) Service—46.9(F), 11.4(M)	Parental Occupational Status (%)	Unemployed—14.5(F), 47(M) Part-time workers—6.4(F), 5.5(M) Service—79.1(F), 47.5(M)
Student—100 Part-time Work—1.5	Participant’s Occupation (%)	Student—100 Part-time Work—32.2

**Table 7:** Simple mediation analysis using PROCESS Macro (mediator variable: potency).

		Effect	SE	t	*p*	LLCI	ULCI	c cs
Total effect of X on Y		0.193	0.021	9.339	0.000	0.152	0.233	0.553
Direct effect of X on Y		0.103	0.023	4.417	0.000	0.057	0.149	0.296
		Effect	BootSE	BootLLCI	BootULCI			
Indirect effect of X on Y	Potency	0.089	0.017	0.058	0.126			
Completely standardized indirect effect of X on Y	Potency	0.258	0.044	0.175	0.348			

X = predictor variable = perception of poverty, Y = dependent variable = somatisation tendency.

## Data Availability

Data supporting the reported results can be obtained upon request from the corresponding author. The data is not publicly available because of confidentiality issues.

## References

[1] Yates W.R., Dunayevich E. (2014). Medscape—Somatic Symptom Disorders. https://emedicine.medscape.com/article/294908-overview.

[2] Rhee H., Miles M.S., Halpern C.T., Holditch-Davis D. (2005). Prevalence of recurrent physical symptoms in US adolescents. Pediatr. Nurs..

[3] Swain M.S., Henschke N., Kamper S.J., Gobina I., Ottová-Jordan V., Maher C.G. (2014). An international survey of pain in adolescents. BMC Public Health.

[4] Viernes N., Zaidan Z.A., Dorvlo A.S., Kayano M., Yoishiuchi K., Kumano H., Kuboki T., Al-Adawi S. (2007). Tendency toward deliberate food restriction, fear of fatness and somatic attribution in cross-cultural samples. Eat. Behav..

[5] Cheng Q., Xu Y., Xie L., Hu Y., Lv Y. (2019). Prevalence and environmental impact factors of somatization tendencies in eastern Chinese adolescents: A multicenter observational study. Cad. Saude Publica.

[6] Kirmayer L.J., Taillefer S., Hersen M., Turner S. (1997). Somatoform disorders. Adult Psychopathology.

[7] Hobfoll S.E. (2002). Social and psychological resources and adaptation. Rev. Gen. Psychol..

[8] OECD (2018). Education at a Glance 2014: OECD Indicators.

[9] Patel N. (2018). Understanding Psychological Conflicts in Patients with Essential Hypertension and Exploring Matching Homoeopathic Remedies. Homœopath. Links.

[10] Dimsdale J.E., Creed F., Escobar J., Sharpe M., Wulsin L., Barsky A., Lee S., Irwin M.R., Levenson J. (2013). Somatic symptom disorder: An important change in DSM. J. Psychosom. Res..

[11] Dijkstra-Kersten S.M., Sitnikova K., van Marwijk H.W., Gerrits M.M., van der Wouden J.C., Penninx B.W., van der Horst H.E., Leone S.S. (2015). Somatisation as a risk factor for incident depression and anxiety. J. Psychosom. Res..

[12] Creed F.H., Davies I., Jackson J., Littlewood A., Chew-Graham C., Tomenson B., Macfarlane G., Barsky A., Katon W., McBeth J. (2012). The epidemiology of multiple somatic symptoms. J. Psychosom. Res..

[13] Lee S., Creed F.H., Ma Y.L., Leung C.M. (2015). Somatic symptom burden and health anxiety in the population and their correlates. J. Psychosom. Res..

[14] Ruchkin V., Schwab-Stone M. (2014). A longitudinal study of somatic complaints in urban adolescents: The role of internalizing psychopathology and somatic anxiety. J. Youth Adolesc..

[15] Bae S.M., Kang J.M., Chang H.Y., Han W., Lee S.H. (2018). PTSD correlates with somatization in sexually abused children: Type of abuse moderates the effect of PTSD on somatization. PLoS ONE.

[16] Raffagnato A., Angelico C., Valentini P., Miscioscia M., Gatta M. (2020). Using the Body When There Are No Words for Feelings: Alexithymia and Somatization in Self-Harming Adolescents. Front. Psychiatry.

[17] Karkhanis D.G., Winsler A. (2016). Somatization in children and adolescents: Practical implications. J. Indian Assoc. Child Adolesc. Ment. Health.

[18] Frenkel L., Swartz L., Bantjes J. (2018). Chronic traumatic stress and chronic pain in the majority world: Notes towards an integrative approach. Crit. Public Health.

[19] Bourdillon M., Boyden J. (2014). Growing Up in Poverty: Findings from Young Lives.

[20] Brando N., Schweiger G. (2019). Philosophy and Child Poverty: Reflections on the Ethics and Politics of Poor Children and Their Families.

[21] Marmot M., Allen J., Bell R., Bloomer E., Goldblatt P. (2012). WHO European review of social determinants of health and the health divide. Lancet.

[22] Kirmayer L.J., Young A. (1998). Culture and somatization: Clinical, epidemiological, and ethnographic perspectives. Psychosom. Med..

[23] American Psychiatric Association (2013). Diagnostic and Statistical Manual of Mental Disorders.

[24] Hughes M., Tucker W. (2018). Poverty as an adverse childhood experience. North Carol. Med. J..

[25] Shonkoff J.P., Garner A.S., Siegel B.S., Dobbins M.I., Earls M.F. (2012). Technical Report: The lifelong effects of early childhood adversity and toxic stress. From The committees on psychosocial aspects of child and family health; early childhood, adoption and dependent care, and section on developmental and behavioral pediatrics. Pediatrics.

[26] Babu A.R., Aswathy Sreedevi A.J., Krishnapillai V. (2019). Prevalence and determinants of somatization and anxiety among adult women in an urban population in Kerala. Indian J. Community Med..

[27] Gureje O., Simon G.E., Ustun T.B., Goldberg D.P. (1997). Somatization in cross-cultural perspective: A World Health Organization study in primary care. Am. J. Psychiatry.

[28] Wagle U.R. (2006). Poverty in Kathmandu: What do subjective and objective economic welfare concepts suggest?. J. Econ. Inequal..

[29] Blackorby C., Donaldson D. (1987). Welfare ratios and distributionally sensitive cost-benefit analysis. J. Public Econ..

[30] Rank M.R., Hirschl T.A. (2015). The likelihood of experiencing relative poverty over the life course. PLoS ONE.

[31] Lee K., Zhang L. (2022). Cumulative Effects of Poverty on Children’s Social-Emotional Development: Absolute Poverty and Relative Poverty. Community Ment. Health J..

[32] Sen A.K. (1985). Commodities and Capabilities.

[33] Sen A.K. (1999). Development as Freedom.

[34] Cook J.A., Mueser K.T. (2016). Is recovery possible outside the financial mainstream?. Psychiatr. Rehabil. J..

[35] Sylvestre J., Notten G., Kerman N., Polillo A., Czechowki K. (2018). Poverty and serious mental illness: Toward action on a seemingly intractable problem. Am. J. Community Psychol..

[36] Joychan S., Kazi R., Patel D.R. (2016). Psychosomatic pain in children and adolescents. J. Pain Manag..

[37] Petanidou D., Giannakopoulos G., Tzavara C., Dimitrakaki C., Kolaitis G., Tountas Y. (2014). Adolescents’ multiple, recurrent subjective health complaints: Investigating associations with emotional/behavioural difficulties in a cross-sectional, school-based study. Child Adolesc. Psychiatry Ment. Health.

[38] Ran L., Wang W., Ai M., Kong Y., Chen J., Kuang L. (2020). Psychological resilience, depression, anxiety, and somatization symptoms in response to COVID-19: A study of the general population in China at the peak of its epidemic. Soc. Sci. Med..

[39] Keller C.J. (2016). Courage, Psychological Well-being, and Somatic Symptoms. Ph.D. Thesis.

[40] DeLongis A., Folkman S., Lazarus R.S. (1988). The impact of daily stress on health and mood: Psychological and social resources as mediators. J. Personal. Soc. Psychol..

[41] Masten A.S. (2014). Global Perspectives on Resilience in Children and Youth. Child. Dev..

[42] Overton W.F. (2013). A new paradigm for developmental science: Relationism and relational-developmental systems. App. Dev. Sci..

[43] Wright M.O.D., Masten A.S., Narayan A.J., Goldstein S., Brooks R.B. (2013). Resilience processes in development: Four waves of research on positive adaptation in the context of adversity. Handbook of Resilience in Children.

[44] Ben-Sira Z. (1985). Potency: A stress-buffering link in the coping-stress-disease relationship. Sot. Sci. Med..

[45] Lev-Wiesel R. (2009). Enhancing potency among male adolescents at risk to drug abuse: An action research. Child Adolesc. Soc. Work. J..

[46] Bhattacharyya A., Lev-Wiesel R., Banerjee M. (2020). Roles of Emotional Reactions and Potency in Coping with Abusive Experiences of Indian Adolescent. J. Child Adolesc. Trauma.

[47] Lev-Wiesel R. (1999). Living under the threat of relocation: Different buffering effects of personal coping resources on men and women. Marriage Fam. Rev..

[48] Ladipo M.M., Obimakinde A.M., Irabor A.E. (2015). Familial and socio-economic correlates of somatisation disorder. Afr. J. Prim. Health Care Fam. Med..

[49] Wethington E., Glanz K., Schwartz M.D. (2015). Stress, coping, and health behavior. Health Behav. Theory Res. Pract..

[50] Muñoz L.R. (2020). Graduate student self-efficacy: Implications of a concept analysis. J. Prof. Nurs..

[51] Wallston K.A. (2015). Control Beliefs: Health Perspectives. International Encyclopedia of the Social & Behavioral Sciences.

[52] Ross C.E., Mirowsky J. (2015). Alienation: Psychosociological Tradition. International Encyclopaedia of the Social & Behavioral Sciences.

[53] Deflem M., Triplett R.A. (2018). Anomie, Strain, and Opportunity Structure. The Handbook of the History and Philosophy of Criminology.

[54] The World Bank Data by Country (Israel). http://data.worldbank.org/country/Israel.

[55] The World Bank Data by Country (India). http://data.worldbank.org/country/india.

[56] World Population Review Median Income by Country 2022. https://worldpopulationreview.com/country-rankings/median-income-by-country.

[57] The World Bank, Poverty & Equity Data Portal—India. http://povertydata.worldbank.org/poverty/country/IND.

[58] Harel-Shalev A. (2009). Lingual and educational policy toward “Homeland Minorities” in deeply divided societies: India and Israel as case studies. Politics Policy.

[59] Bhandari L., Chakraborty M. (2015). Spatial Poverty in West Bengal. https://www.livemint.com/Opinion/dXPv8bp492mKX9rirXX0hK/Spatial-poverty-in-West-Bengal.html.

[60] Tel-Aviv Planning and Development Department. https://www.tel-aviv.gov.il/en/Pages/HomePage.aspx.

[61] Kolkata Population. http://www.census2011.co.in/census/city/215-kolkata.html.

[62] World Data.info Country Comparison. https://www.worlddata.info/countrycomparison.php?country1=IND&country2=ISR.

[63] Ben-Ezra M., Karatzias T., Hyland P., Brewin C.R., Cloitre M., Bisson J.I., Roberts N.P., Lueger-Schuster B., Shevlin M. (2018). Posttraumatic stress disorder (PTSD) and complex PTSD (CPTSD) as per ICD-11 proposals: A population study in Israel. Depress. Anxiety.

[64] Smartt C., Medhin G., Alem A., Patel V., Dewey M., Prince M., Hanlon C. (2016). Fatigue as a manifestation of psychosocial distress in a low-income country: A population-based panel study. Trop. Med. Int. Health.

[65] Wagle U.R. (2010). Does Low Inequality Cause Low Poverty? Evidence from High-Income and Developing Countries. Poverty Public Policy.

[66] Ravallion M., Chen S. (2011). Weakly relative poverty. Rev. Econ. Stat..

[67] BBC News UK Where Are You on the Global Pay Scale. http://wwwnews.live.bbc.co.uk/news/magazine-17543356.

[68] Anand S., Segal P., Atkinson A.B., Bourguignon F. (2015). The global distribution of income. Handbook of Income Distribution.

[69] Lafrance R., Schembri L.L. (2002). Purchasing-power parity: Definition, measurement, and interpretation. Bank Can. Rev..

[70] Brislin R. (1970). Back translation for cross-cultural research. J. Cross Cult. Psychol..

[71] Brislin R., Triandis H.C., Berry J.W. (1980). Translation and content analysis of oral and written materials. Handbook of Cross-Cultural Psychology: Methodology.

[72] Hui C.H., Triandis H.C. (1985). Measurement in cross-cultural psychology a review and comparison of strategies. J. Cross Cult. Psychol..

[73] Mumford D.B., Bavington J.T., Bhatnagar K.S., Hussain Y., Mirza S., Naraghi M.M. (1991). The Bradford Somatic Inventory: A multi-ethnic inventory of somatic symptoms reported by anxious and depressed patients in Britain and the Indo-Pakistan subcontinent. Br. J. Psychiatry.

[74] Halik M., Webley P. (2011). Adolescents’ understanding of poverty and the poor in rural Malaysia. J. Econ. Psychol..

[75] Nunnally J.C., Bernstein I.H. (1994). Psychological Theory.

[76] Haynes S.N., Richard D., Kubany E.S. (1995). Content validity in psychological assessment: A functional approach to concepts and methods. Psychol. Assess..

[77] Kohli A.K., Zaltman G. (1988). Measuring multiple buying influences. Ind. Mark. Manag..

[78] Field A.P. (2009). Discovering Statistics Using SPSS: And Sex and Drugs and Rock ‘N’ Roll.

[79] Kleinbaum D.G., Kupper L.L., Morgenstern H. (1982). Epidemiologic Research: Principles and Quantitative Methods.

[80] Maldonado G., Greenland S. (1993). Simulation study of confounder-selection strategies. Am. J. Epidemiol..

[81] Kutner M.H., Nachtsheim C., Neter J. (2004). Applied Linear Regression Models.

[82] Memon M.A., Jun H.C., Ting H., Francis C.W. (2018). Mediation analysis issues and recommendations. J. Appl. Struct. Equ. Model..

[83] Preacher K.J., Hayes A.F. (2004). SPSS and SAS procedures for estimating indirect effects in simple mediation models. Behav. Res. Methods Instrum. Comput..

[84] Chander K.R., Manjunatha N., Binukumar B., Kumar C.N., Bada Math S., Janardhan Reddy Y. (2019). The prevalence and its correlates of somatization disorder at a quaternary mental health centre. Asian J. Psychiatry.

[85] Escobar J.I., Cook B., Chen C.N., Gara M.A., Alegría M., Interian A., Diaz E. (2010). Whether medically unexplained or not, three or more concurrent somatic symptoms predict psychopathology and service use in community populations. J. Psychosom. Res..

[86] Shechory Bitton M., Laufer A. (2018). Children’s emotional and behavioral problems in the shadow of terrorism: The case of Israel. Child. Youth Serv. Rev..

[87] Stein J.Y., Levin Y., Gelkopf M., Tangir G., Solomon Z. (2017). Traumatization or Habituation? A Four-wave Investigation of Exposure to Continuous Traumatic Stress in Israel. Int. J. Stress Manag..

[88] Kirmayer L.J., Swartz L. (2013). Culture and global mental health. Global Mental Health: Principles and Practice.

[89] Slone M., Lavi I., Ozer E.J., Pollak A. (2017). The Israeli-Palestinian conflict: Meta-analysis of exposure and outcome relations for children of the region. Child. Youth Serv. Rev..

[90] Rosshandler Y., Hall B.J., Canetti D. (2016). An application of an ecological framework to understand risk factors of PTSD due to prolonged conflict exposure: Israeli and Palestinian adolescents in the line of fire. Psychol. Trauma Theory Res. Pract. Policy.

[91] Slone M., Shoshani A., Lobel T. (2013). Helping youth immediately following war exposure: A randomized controlled trial of a school-based intervention program. J. Prim. Prev..

[92] Shelef L., Dotan S., Kaminsky D., Kedem R., Margulis A., Hassidim A. (2016). Relationship between anxiety and medical disorders among compulsory military service candidates between the years 1998–2013. Psychiatry Res..

[93] Fink P., Schröder A. (2010). One single diagnosis, bodily distress syndrome, succeeded to capture 10 diagnostic categories of functional somatic syndromes and somatoform disorders. J. Psychosom. Res..

[94] Morina N., Kuenburg A., Schnyder U., Bryant R.A., Nickerson A., Schick M. (2018). The Association of Post-traumatic and Postmigration Stress with pain and other somatic symptoms: An explorative analysis in traumatized refugees and asylum seekers. Pain Med..

[95] Iffland B., Sansen L.M., Catani C., Neuner F. (2014). Rapid heartbeat, but dry palms: Reactions of heart rate and skin conductance levels to social rejection. Front. Psychol..

[96] Liedl A., O’donnell M., Creamer M., Silove D., McFarlane A., Knaevelsrud C., Bryant R.A. (2010). Support for the mutual maintenance of pain and post-traumatic stress disorder symptoms. Psychol. Med..

[97] Hinton D.E., Kredlow M.A., Pich V., Bui E., Hofmann S.G. (2013). The relationship of PTSD to key somatic complaints and cultural syndromes among Cambodian refugees attending a psychiatric clinic: The Cambodian Somatic Symptom and Syndrome Inventory (CSSI). Transcult. Psychiatry.

[98] Eisenberger N.I. (2012). The pain of social disconnection: Examining the shared neural underpinnings of physical and social pain. Nat. Rev. Neurosci..

[99] Afari N., Ahumada S.M., Wright L.J., Mostoufi S., Golnari G., Reis V., Cuneo J.G. (2014). Psychological trauma and functional somatic syndromes: A systematic review and meta-analysis. Psychosom. Med..

[100] Bøe T., Petrie K.J., Sivertsen B., Hysing M. (2019). Interplay of subjective and objective economic well-being on the mental health of Norwegian adolescents. SSM Popul. Health.

[101] Chang Q., Peng C., Guo Y., Cai Z., Yip P.S. (2020). Mechanisms connecting objective and subjective poverty to mental health: Serial mediation roles of negative life events and social support. Soc. Sci. Med..

[102] Murali V., Oyebode F. (2004). Poverty, social inequality and mental health. Adv. Psychiatr. Treat..

[103] Maguire-Jack K., Yoon S., Hong S. (2022). Social Cohesion and Informal Social Control as Mediators Between Neighborhood Poverty and Child Maltreatment. Child Maltreat..

[104] Pudrovska T., Schieman S., Pearlin L.I., Nguyen K. (2005). The sense of mastery as a mediator and moderator in the association between economic hardship and health in late life. J. Aging Health.

[105] Lever J.P., Piñol N.L., Uralde J.H. (2005). Poverty, psychological resources and subjective well-being. Soc. Indic. Res..

[106] Bandura A. (1989). Human agency in social cognitive theory. Am. Psychol..

[107] Bovier P.A., Chamot E., Perneger T.V. (2004). Perceived stress, internal resources, and social support as determinants of mental health among young adults. Qual. Life Res..

[108] Grant K.E., Katz B.N., Thomas K.J., O’Koon J.H., Meza C.M., DiPasquale A.M., Rodriguez V.O., Bergen C. (2004). Psychological symptoms affecting low-income urban youth. J. Adolesc. Res..

[109] Lorincová T., Lelková A. (2016). Prediction of manipulation, empathy and social irritability through selected personality traits among managers. Period. Polytech. Soc. Manag. Sci..

[110] Delamater J.D., Myers J.M. (2010). Social Psychology.

[111] Hofstede G. (1980). Culture’s Consequences: International Differences in Work-Related Values.

[112] Hofstede G. (2001). Culture’s Consequences: Comparing Values, Behaviors, Institutions and Organizations Across Nations.

[113] Bleidorn W., Arslan R.C., Denissen J.J., Rentfrow P.J., Gebauer J.E., Potter J., Gosling S.D. (2016). Age and gender differences in self-esteem—A cross-cultural window. J. Personal. Soc. Psychol..

[114] Gozansky T. (2014). Between Expropriation and Exploitation: Status and Struggles of Arab Workers in Palestine and Israel.

[115] Margalioth S.R. (2004). Labor market discrimination against Arab Israeli citizens: Can something be done. N. Y. Univ. J. Int. Law Polit..

[116] Ben-Sira Z. (1984). Chronic illness, stress and coping. Soc. Sci. Med..

[117] Fohrbeck A., Hirseland A., Lobato P.R. (2014). How benefits recipients perceive themselves through the lens of the mass media-some observations from Germany. Sociol. Res. Online.

[118] Schweiger G., Schweiger G. (2020). Disappointed Expectations. On Misrecognition and Poverty. Poverty, Inequality and the Critical Theory of Recognition. Philosophy and Poverty.

[119] Parikh P., Fu K., Parikh H., McRobie A., George G. (2015). Infrastructure provision, gender, and poverty in Indian slums. World Dev..

[120] Majumder R. (2010). Intergenerational mobility in educational and occupational attainment: A comparative study of social classes in India, Margin. J. Appl. Econ. Res..

[121] Nwokocha A.R., Chinawa J.M., Onukwuli V., Ubesie A., Ndukuba A., Chinawa A.T., Aniwada E., Uwaezuoke S. (2017). Somatization disorder among adolescents in southeast Nigeria: A neglected issue. Int. J. Ment. Health Syst..

[122] Cummings C.M., Caporino N.E., Kendall P.C. (2014). Comorbidity of anxiety and depression in children and adolescents: 20 years after. Psychol. Bull..

[123] Prabhu V. (2010). Tests of intra household resource allocation using a CV framework: A comparison of husbands’ and wives’ separate and joint WTP in the slums of Navi-Mumbai, India. World Dev..

[124] World Bank (WB) (2006). World Development Report: Equity and Development.

[125] Dercon S., Singh A. (2013). From nutrition to aspirations and self efficacy: Gender bias over time among children in four countries. World Dev..

[126] Floro M., Swain R. (2013). Food security, gender, and occupational choice among urban low-income households. World Dev..

[127] Maitra S., Brault M.A., Schensul S.L., Schensul J.J., Nastasi B.K., Verma R.K., Burleson J.A. (2015). An approach to mental health in low-and middle-income countries: A case example from urban India. Int. J. Ment. Health.

[128] Patel V., Araya R., De Lima M., Ludermir A., Todd C. (1999). Women, poverty and common mental disorders in four restructuring societies. Soc. Sci. Med..

[129] Evans G.W., English K. (2002). The environment of poverty: Multiple stressor exposure, psychophysiological stress, and socioemotional adjustment. Child Dev..

[130] Karvonen S., Rahkonen O. (2011). Subjective social status and health in young people. Sociol. Health Illn..

[131] Jeon G.S., Ha Y., Choi E. (2013). Effects of objective and subjective socioeconomic status on self-rated health, depressive symptoms, and suicidal ideation in adolescents. Child Indic. Res..

[132] Reynolds L.K., O’koon J.H., Papademetriou E., Szczygiel S., Grant K.E. (2001). Stress and somatic complaints in low-income urban adolescents. J. Youth Adolesc..

